# Directions for new developments on statistical design and analysis of small population group trials

**DOI:** 10.1186/s13023-016-0464-5

**Published:** 2016-06-14

**Authors:** Ralf-Dieter Hilgers, Kit Roes, Nigel Stallard, for the IDeAl, Asterix and InSPiRe project groups, Corinne Alberti, Caroline van Baal, Norbert Benda, Egbert Biesheuvel, Carl Fredrik Burmann, Malgorzata Bogdan, Emmanuelle Comets, Simon Day, Holger Dette, Alex Dmitrienko, Tim Friede, Alexandra Graf, Mats Karlsson, Armin Koch, Franz König, Johanna H. van der Lee, Frederike Lentz, Jason Madan, Christoph Male, France Mentré, Frank Miller, Geert Molenberghs, Beat Neuenschwander, Martin Posch, Cor Oosterwijk, Christian Röver, Stephen Senn, Ferran Torres, Sarah Zohar

**Affiliations:** Department of Medical Statistics, RWTH Aachen University, Pauwelsstr 30, D-52074 Aachen, Germany; Quality and Patient Safety, Biostatistics, UMC Utrecht, Utrecht, Netherlands; Statistics and Epidemiology, Warwick Medical School, University of Warwick, Coventry, UK

**Keywords:** EMA/CHMP Guideline on clinical trials in small populations, Statistical methods, Statistical design, Statistical analysis, Small population clinical trials, Rare disease

## Abstract

**Background:**

Most statistical design and analysis methods for clinical trials have been developed and evaluated where at least several hundreds of patients could be recruited. These methods may not be suitable to evaluate therapies if the sample size is unavoidably small, which is usually termed by small populations. The specific sample size cut off, where the standard methods fail, needs to be investigated. In this paper, the authors present their view on new developments for design and analysis of clinical trials in small population groups, where conventional statistical methods may be inappropriate, e.g., because of lack of power or poor adherence to asymptotic approximations due to sample size restrictions.

**Method:**

Following the EMA/CHMP guideline on clinical trials in small populations, we consider directions for new developments in the area of statistical methodology for design and analysis of small population clinical trials. We relate the findings to the research activities of three projects, Asterix, IDeAl, and InSPiRe, which have received funding since 2013 within the FP7-HEALTH-2013-INNOVATION-1 framework of the EU. As not all aspects of the wide research area of small population clinical trials can be addressed, we focus on areas where we feel advances are needed and feasible.

**Results:**

The general framework of the EMA/CHMP guideline on small population clinical trials stimulates a number of research areas. These serve as the basis for the three projects, Asterix, IDeAl, and InSPiRe, which use various approaches to develop new statistical methodology for design and analysis of small population clinical trials. Small population clinical trials refer to trials with a limited number of patients. Small populations may result form rare diseases or specific subtypes of more common diseases. New statistical methodology needs to be tailored to these specific situations.

**Conclusion:**

The main results from the three projects will constitute a useful toolbox for improved design and analysis of small population clinical trials. They address various challenges presented by the EMA/CHMP guideline as well as recent discussions about extrapolation. There is a need for involvement of the patients’ perspective in the planning and conduct of small population clinical trials for a successful therapy evaluation.

## Background

Most statistical design and analysis methods for clinical trials have been developed in the setting of confirmatory trials with relatively large sample sizes, perhaps several hundreds or even thousands of patients. These methods may not be suitable to evaluate therapies in small populations, for example when the size of the population is limited because a disease is rare, or a treatment is targeted at a particular genetic subgroup or a small paediatric population. Such cases, where there is a limited number of patients that could potentially be enrolled in the trial, raise a number of specific statistical challenges and can lead to slow approval of orphan drugs for marketing and poorly designed studies [1].

### The Asterix, IDeAl and InSPiRe projects

In the light of these challenges, in 2013 the European Commission set up a unique call for new methodologies for clinical trials for small population groups within the FP7-HEALTH-2013-INNOVATION-1 [2] framework. This builds on other initiatives from around the world aimed at improving research, including methodology, in rare diseases [3]. The objective of the research is to develop new or improved statistical methodologies for clinical trials for the assessment of treatments for small population groups, in particular for rare diseases or personalised (stratified or individualised) medicine. Research is expected to be multidisciplinary and should involve all relevant stakeholders including industry and patient advocacy groups as appropriate.

**Fig. 1:**
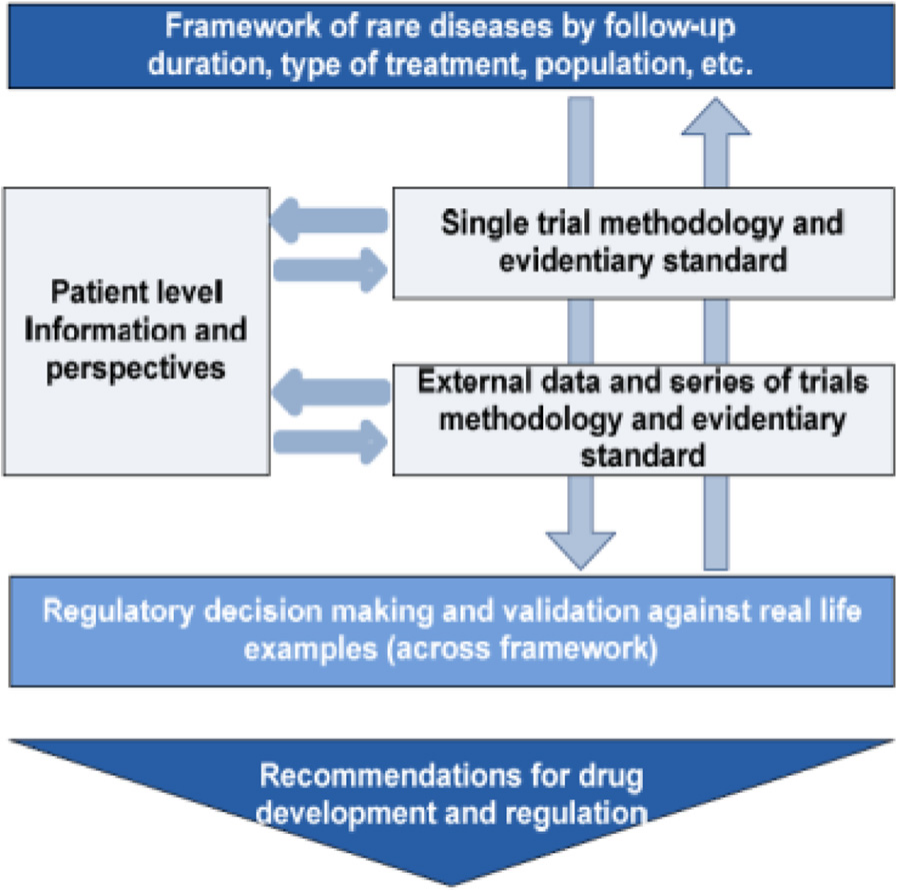
Asterix approach to advance trial design in small populations

**Fig. 2:**
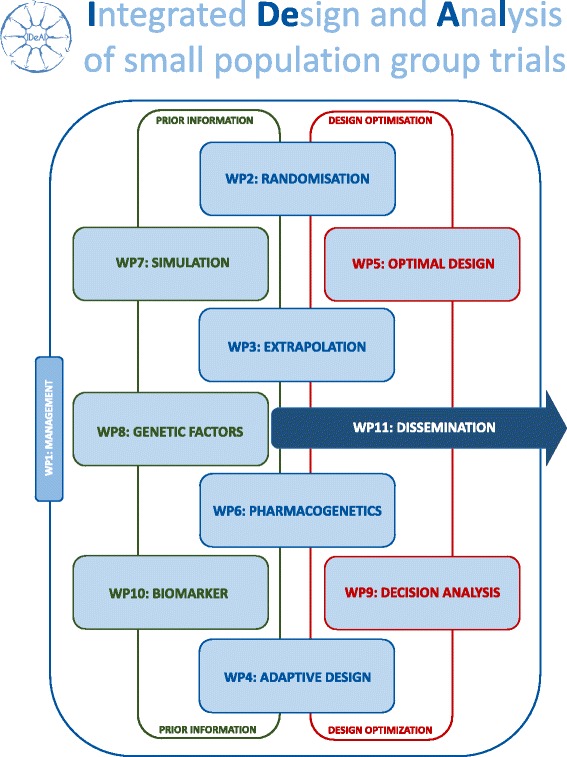
Exhibit of the IDeAl project broken down in the workpackages

**Fig. 3:**
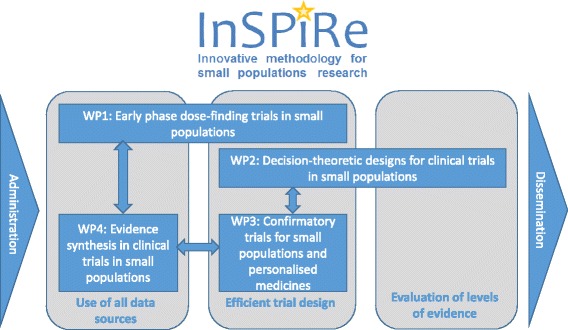
Exhibit of the InSpiRe project broken down in the work packages

Three collaborative research projects Asterix, IDeAl, and InSPiRe, are funded under this call. As illustrated in Figs. 1, 2 and 3, the three projects focus on a number of methodological challenges in the design, analysis and interpretation of clinical trials in small populations and rare diseases as well as considering specific aspects such as patient perspectives and ethical issues.

### Regulatory guidance

In the EU, a disease is defined as rare if the prevalence is not more than 5 in 10,000. New drug treatments for these rare diseases may be eligible for orphan drug designation, also depending on other conditions – such as the status of existing treatments. The number of rare diseases is estimated to be around 7000 [4]. Thus although the diseases themselves are rare, the total number affected in the EU is estimated to exceed 30 million [5] with 6 % of the global population affected by a rare disease at some stage in their life [6], resulting in considerable total health care cost [7]. More than 1120 *orphan drug designations* have been granted by the EMA since 2000, and 114 orphan drugs received market authorization by January 2016 [8]. This seem to be small compared to the objective of the international rare disease research consortium to develop 200 new therapies by 2020 [9]. Although many factors complicate the development of new medicines for orphan diseases, the main issue setting it apart from drug development for common diseases is the challenge of generating acceptable evidence from clinical trials in the clinical research phase in which recruitment is necessarily limited.

In the EU clinical trials in drug development for small population groups should take into account the EMA/CHMP guideline [10] together with a recently published reflection paper on extrapolation of efficacy and safety in medicine development [11]. In the US the FDA drafted guideline on orphan drug approval has been published [12], whereas in Japan no such specific guidance for clinical trials in small populations exists. To summarize, the EMA/CHMP guideline states, that “No methods exist that are relevant to small studies that are not also applicable to large studies. However, it may be that in conditions with small and very small populations, less conventional and/or less commonly seen methodological approaches may be acceptable if they help to improve the interpretability of the study results” [10]. Further it is recommended to use as much information as possible for designing a clinical trial and extract as much information as possible from a clinical trial to enable valid benefit risk assessment. Additionally the EMA/CHMP guideline [11] suggests avoiding unnecessary clinical trials e.g., by extrapolation from a larger source population to a smaller target population when this is appropriate.

### Paper outline

This paper is structured around the headings in the EMA/CHMP guideline [10]; “Levels of evidence”, “Pharmacological considerations”, “Choice of endpoints” and “Methodological and statistical considerations”. We add “extrapolation” and “patient involvement and ethical considerations” as additional points, but do not consider specifically “Choice of control groups” except when this arises in association with the other headings. Under each heading we give a brief overview of the key statistical and methodological challenges and explain how the three EU funded projects Asterix, IDeAl, and InSPiRe address these challenges and will stimulate uptake of new methodology in practice. Some further aspects will be considered, where we feel that extensions are helpful, e.g., taking into account health economic aspects.

## Biostatistical Research by Asterix, IDeAl and InSPiRe

### Levels of evidence

The EMA/CHMP guideline states that, as a general rule, the same standards of levels of evidence are to be applied to applications for marketing authorisations in small populations as are used for other products. The usual hierarchy of evidence places evidence from randomised controlled trials, either individual trials or meta-analyses of trials, as being of the highest quality. Conventionally, sample sizes for definitive randomised controlled trials are large and in some small population settings, particularly in very rare diseases, such trials may be infeasible. The guideline indicates that the limitation on patient recruitment in a small population setting will be taken into account. The level of evidence required might thus partly be determined by the potential availability of evidence, with decisions taken accordingly. From a regulatory perspective, exceptions from the default licensing requirements are considered in the interest of patients’ access to treatments. ‘Marketing Authorisation under Exceptional Circumstances’ permits licensing under greater than usual uncertainty when a ‘comprehensive clinical evidence base’ is not achievable. This, however, leads to case-by-case decision making, which makes planning for prospective drug development strategies more difficult, and hampers adhering to the same standard. Hence, providing a more quantitative foundation for the level of evidence applied is an important challenge. This will be addressed by the research of the Asterix, IDeAl and InSPiRe projects as follows:

- An approach will be developed based on Bayesian decision theory to obtain optimised clinical trial designs that account for prior belief regarding the benefits of treatment and the population size.
- Methods to relax the traditionally applied levels of type 1 and type 2 errors based on the anticipated finite (and relatively small) future population to treat will be developed, essentially based on a mix of Bayesian and frequentist methods.
- Value of information methods will be applied to assess appropriate sample sizes for clinical trials in small populations.
- In small clinical trials properties of randomization procedure relying on approximate arguments fail to suffice. Recommendations for selection of the best practice randomization procedure maintaining the significance level in the presence of selection bias will be derived.
- Determination of appropriate levels of evidence for decision-making in small population clinical trials will be investigated.

When evidence from randomised controlled trials is limited or absent, evidence from lower levels in the hierarchy, including observational studies or case-reports, may become of greater importance together with systematic review and synthesis of all available information, whatever its source. The formal synthesis of evidence from sources of differing quality, briefly evidence synthesis, using either Bayesian or frequentist meta analysis methods, can present particular statistical challenges.

This challenge will be addressed by the research of the Asterix, IDeAl, and InSPiRe projects in the following ways:

- Evidence synthesis methods for small populations and rare diseases will be developed to support the planning, analysis and interpretation of a single randomised controlled trial. The feasibility and utility of the newly developed methods will be assessed in small population settings.
- Generalized evidence synthesis approaches will be applied to paediatric studies and compounds developed for potentially multiple rare indications.
- Evidence synthesis methods across trials (of similar or different design) that take into account the sequential nature of drug development will be developed.

### Pharmacological considerations

The “pharmacological considerations” section of the EMA/CHMP guideline suggests that the design of a clinical trial could be improved by detailed pharmacological knowledge of the disease and the drug under consideration [13]. As elaborated in the guideline, knowledge about the variability is essential for efficient study design and use of the best available techniques to obtain and analyse information is crucial. It is well known that poor designs may be a unnecessary source of variation.

Many drugs are metabolized in a nonlinear e.g., exponential fashion. Thus the description of pharmacological processes needs the application of complex statistical models, such as non-linear mixed effects models. These models allow the evaluation of drug profiles resulting from repeated drug concentration measurements from a group of patients while considering individual variation. As these models are extensions of the usual linear mixed effects models, they are nonlinear in the parameters to be estimated, which complicates the optimal design problem.

These aspects will be addressed by the research in the Asterix, IDeAl, and InSPiRe projects in the following way:

- Pharmacometric [14] approaches will be considered by modelling the disease – therapy relation using non-linear mixed effect models. These theoretical models are used for improved sample size estimation in small clinical trials allowing for uncertainty, defining appropriate outcome measures and investigating analysis methods.
- Optimal design techniques, resulting in time points of observation based on minimization of the variance of the effects estimators in nonlinear mixed-effects models will be developed by means of using preliminary estimates from interim looks to improve the final estimator. New numerical techniques in case of small samples where asymptotic assumptions are no longer valid.
- Innovative designs for early phase clinical trials taking into account safety, efficacy and pharmacokinetic/pharmacodynamic (PK/PD) measures will be developed to better estimate the dose level to be recommended based on limited sample sizes and subgroups with continuous and binary outcomes.
- The performance of the novel methods will be evaluated in terms of information gain, number of subjects, efficiency, and robustness. Designs for within patient data modelling will be developed to allow as much information as possible to be used for benefit risk assessment.
- Improved methods for identification of genetic prognostic factors will be derived, leading to efficient clinical trial design and analysis.

### Choice of endpoints

The EMA/CHMP guideline indicates that although hard clinical endpoints, such as survival or serious mobidity, are preferred, surrogate endpoints may be an option in small populations clinical trials [10] although evaluation of the surrogates might be challenging [15]. Another direction in particular in small clinical trials is to use endpoints from medical care which are important for the patients, briefly patient-centered endpoints [16], e.g., where hard clinical endpoints (such as survival) cannot be observed in sufficient patients within a reasonable timeframe. This implies further challenges like showing the clinical relevance of the patient-centered endpoint, capacity to measure the risk-benefit relation and reliability considerations. On the other hand, the problem of how to derive a uniform definition of a patient-centered endpoint has not yet been solved. Due to clinical heterogeneity of the disease course in many rare diseases, patients’ need for improvement can vary substantially with respect to outcome measure. Although patient involvement in the definition of outcomes is high on the agenda of public funders, regulators and international research groups [17, 18], many generic measurement instruments for patient-centered outcomes may not be sufficiently responsive to detect changes for a particular disease. Further, the primary analysis of most confirmatory clinical trials relies on a single primary endpoint, which does not reflect the need to use as much information as possible in particular in small clinical trials where typically a range of relevant outcome measures describes the benefit/risk of a treatment. Making use of this information may improve efficiency by capturing heterogeneity between patients’ disease courses. Related to this is the heterogeneous and multivariate nature of the disease course, where it may not be obvious which aspects are actually affected by treatment or need priority. These issues will be addressed in the three EU funded projects as follows:

- A suitable framework for validation of biomarkers as surrogate endpoints in small clinical trials will be developed and optimal designs investigated. Hereby, the most appropriate approach, e.g., the causal inference and meta analytical paradigm will be evaluated within the context of small samples.
- The impact of unbalanced small data sets on numerical accuracy for mixed effects models used for surrogate endpoint validation will be investigated.
- Recommendations for models to describe the reliability of measurements in longitudinal studies, where the number and measurement times differs between subjects and the number of longitudinal measurements is large compared to the number of subjects of the trial will be established.
- The possibility of using an individualized outcome measurement instrument, called Goal Attainment Scale [19], will be investigated. This may enable generation of evidence in diseases for which patient variability precludes the use of conventional variables to demonstrate efficacy.
- Strategies will be developed that use information from multiple endpoints to make inference on treatment benefit [2, 14, 20–23].

### Methodological and statistical considerations

The section on “Methodological and statistical considerations” in the EMA/CHMP guideline covers “design stage,” “data analysis,” and “reporting”. Here it is stated that the sources of variation should be reduced by using efficient designs and the variation should be explained by adequate statistical models. Elements of this, such as the choice of the endpoint and incorporation of exploratory factors in the statistical model, have implications on the precision of the treatment effect estimate and the optimal design choice. General approaches taken to address these issues in the three EU funded projects include:

- Specific designs which are recommended in small population clinical trials will be evaluated concerning, whether it is possible to estimate the treatment effect without bias.
- Once the treatment estimate could be estimated without bias, optimal designs minimizing the variation of the estimates, e.g., in non-linear mixed effects models to analyse data in small population groups will be derived.

Randomisation is a key element in designing a clinical trial in particular to avoid bias. Matching and stratification are mentioned in the EMA/CHMP guideline as options to improve the power, but the effectiveness of stratified randomisation in small clinical trials is questionable. A particular challenge in rare diseases, where recruitment times can be prolonged, are time trend biases that can occur if patients’ response to treatments change over time in some systematic way. Further, selection bias could invalidate study results even in double blind trials, where blinding is subverted by observing adverse events [24]. These problems are more relevant in small clinical trials where restricted randomisation methods are favored by scientists and long run arguments may be invalid. The three EU funded projects will address the following objectives:

- The impact of selection and time trend bias on the validity of trial results depending on the selected randomisation procedure will be investigated and recommendations derived for different endpoints such as continuous or time to event.
- A framework for scientific arguments to select the “best” randomisation procedure reflecting the practical clinical constraints will be derived.
- A bias corrected randomisation test will be developed.
- The advantage of covariate or stratified randomisation procedures in small population clinical trials will be examined.
- The benefits and limitations of response adaptive randomisation in small population clinical trials will be evaluated.

Options for efficient clinical trial design are adaptive or sequential approaches in which data are evaluated at a series of interim analyses used to stop or modify the remainder of a clinical trial [25]. Within this context the three EU funded projects will address the following questions:

- Adaptive designs such as multi-arm multi-stage trials suitable for small populations will be developed.
- Modelling and extrapolation tools will be incorporated in adaptive designs for confirmatory studies.
- Adaptive designs that take into account multiple endpoints, biomarkers or surrogate endpoints efficiently will be developed.
- The use of adaptive designs within a trial will be compared with the use of adaptive strategies across trials in a drug development programme.
- The use of adaptive estimation in non-linear mixed effects models to gain in precision and sample size will be explored.
- The extent of bias due to interim analysis will be investigated.

Special types of study designs are needed if very few patients are affected or if individualised therapies should be evaluated. For instance, the EMA/CHMP guideline includes a discussion of n-of-1 trials, a study design which has found little attention in large population trials. In such trials the patient receives a series of treatments preferably selected in random order for a chronic disease that quickly return to stable baseline values after treatment. These aspects are addressed in the three research projects:

- Pharmacogenetic information for tailored therapeutics including its application in crossover trials, n-of-1 trials and enrichment trials will be investigated.
- Randomisation and bias arguments for the validity of n-of-1 trials will be elaborated and randomisation based inference will be developed.

Aspects of data analysis mentioned in the EMA/CHMP guideline include the distributional assumptions required in the analysis of small clinical trials. The unsatisfactory restriction to use only descriptive statistical methods for analysis of small clinical trials [26] is in contrast to the high amount of information often resulting from longitudinal observations in small clinical trials. Non-parametric analysis and/or statistical modelling of longitudinal data may serve as tools for improved statistical analysis. As the EMA/CHMP guideline states, such methods “while not relying on strong distributional assumptions should be considered and may be preferred compared to parametric statistical approaches when assumptions justifying a population based inference are questionable.” The three EU funded projects will address these points as follows:

- The use of randomisation-based inference in small clinical trials, where asymptotic behaviour of parametric tests is questionable will be evaluated. In particular, randomisation tests for complex statistical models and response adaptive randomisation procedures will be developed

The guideline recommends using prognostic factors as stratification factors in the randomisation and analysis in order to minimize the variance of treatment effect estimates. This consideration leads to the following objectives in the three EU funded projects:

- The gain in efficiency of stratification in small clinical trials will be investigated.
- The use of non-linear and linear mixed effects models for longitudinal, hierarchical, and clustered data in small data sets and for unbalanced data will be considered. In particular, non- or semi-parametric methods that do not rely, or rely less, on asymptotic arguments will be developed.
- More complex models that include genetic factors that influence the response to therapy in small population groups will be studied.
- Longitudinal data analysis methods will be improved with respect to non-parametric methods to overcome potential weaknesses caused by the asymptotic theory.
- Frequentist and decision theoretic methods will be developed to predict patients’ responses to targeted treatments based on genetic features or other biomarkers enabling subgroups of patients for which the benefit risk balance of a treatment is positive to be identified and confirmed.

Bayesian methods, in particular Bayesian designs, may be effective in small clinical trials and lead to increased acceptance in the context of evaluation of medical devices [27]. Certainly FDA has approved some devices for orphan indication based on use of informative priors. However, the introduction of prior belief is often a concern in therapy evaluation. Bayesian ideas may also be used for decision analysis. This requires a multi-disciplinary approach. Such methods are often used in health technology assessment, although the evaluation criteria may vary significantly. The three EU funded projects will address these challenges as follows:

- The decision analysis framework will be expanded by explicitly studying the inter-relationship between the decision analyses made by health technology assessors, regulators, patients and trial sponsors.
- The way in which decision analysis affects the design of a clinical trial will be evaluated.

### Extrapolation

As already stated, clinical trials in small populations are challenging because of the limited number of patients that can be enrolled. One way out of the limitations may be to attempt to extrapolate from clinical trials of similar treatments or of the same treatment in other diseases or larger population groups. It may be helpful for example if a drug is already in use for a more common disease [28] and for diseases that are rare in the EU but more common in other continents. Another aspect of extrapolation concerns the modelling of dose response information by the transfer of knowledge from larger to smaller populations so that it is possible to avoid unnecessary studies [11]. This is reflected by the following objectives of the three EU-funded projects:

- The uncertainty in dose–response information when knowledge of disease and drug is used to extrapolate from a large population to a much smaller target population will be quantified.
- The use of extrapolation within a Bayesian framework will be explored.
- Adaptive design strategies for extrapolation studies will be considered.
- Guidance on the data to include in registries to support extrapolation and control group data for trial design will be developed.

### Patient involvement and ethical considerations

Including the patient perspective into the design of trials is of general interest. Arguably it is even more needed in rare diseases, for which there is an urgent need for the development of new treatments, than in more common diseases for most of which some type of treatment is already available. Patients and their representatives are in a crucial position to influence the willingness to participate in clinical trials. There is no consensus on the best practice of involving patients or patient representatives in the design phase of a trial, let alone a series of trials. In the case of rare diseases this is complicated by the small number, geographical spread and possible clinical heterogeneity of the available participants in such a pre-trial design process. However, the level of patient involvement is usually high – particularly in rare diseases there are many patient representation organizations (in the Orphanet database over 2500 patient organizations in Europe are registered, with over 500 operating at an international level). The three EU funded projects will address the questions in the following ways:

- A “Patient Think Tank” is established within one of the projects to directly involve the patient voice in a project and to work together on models for patient involvement in design stages of a trial. Patient organizations are represented in this Think Tank.
- Involvement of patients at the design stage is also aimed to assess impact of design features (such as assessment schedules, informed consent procedures, multiple treatment arms) on willingness and ability to participate.

Since research in rare diseases primarily remains research with human research subjects, the common framework for ethically evaluating clinical research equally applies to studies with small populations. Innovative trial designs in rare diseases may yield new ethical questions that have to be addressed, such as how we should balance scientific validity and reliability of evidence for favourable benefit-risk ratio. Another example is that informed consent processes may differ if innovative designs like adaptive trials are used [29]. For instance, one may ask whether participants should be able to understand that those who enter an adaptive trial later may have a higher chance of receiving the more favourable treatment. Research questions addressed by the three EU funded projects will include:

- To what extent does our ethical framework need to be adjusted to accommodate using innovative trial designs in the context of research on orphan diseases?
- Could we ethically accept less stringent standards of evidence in the context of rare diseases in order to benefit people with orphan diseases sooner?

Ethical perspectives are reflected in the projects in particular in the extended advisory boards. In answering these questions, the framework of Emmanuel [30] is used as starting point.

### Regulatory impact and dissemination

For regulatory impact it is essential that specific guidance can be given on the use and acceptability of new methods. This is where the current guidance may fall short. As there are several thousands of rare diseases development of disease specific guidelines is impractical. To support regulator impact we propose an intermediate approach based on clustering of diseases sharing similar characteristics that determine similar methodological approaches to their study. While previous approaches have focused on the selection of the best design for a single disease, through algorithms guided on disease and treatment characteristics [31, 32], the aim of our clustering is wider, in the sense of serving as the framework to produce methodological and regulatory recommendations that can be valid not only for a single study or disease, but for the general approach to product development of a group of diseases sharing similar traits.

### Awareness of biostatistical methods

A major goal of all three projects is to raise awareness and to disseminate newly developed statistical methods throughout the scientific community involved in clinical research. We will undertake dissemination activities such as teaching relevant groups and informing the scientific community and general public about the project through our websites (www.asterix-fp7.eu, www.ideal.rwth-aachen.de, www.warwick.ac.uk/inspire). We will publish our findings in peer-reviewed journals and stimulate the process of revision or development of regulatory guidelines as necessary. Information about the newly developed results will be targeted towards all relevant groups, e.g., regulators, patient advocacy groups, industry, the scientific community, etc. Further dissemination activities will include giving information about our methods to patient, families, carers, clinicians, social services, regulators, etc., and training of early stage researchers and other interested parties. Results are discussed on a regular basis with the projects’ Advisory Boards that include representatives from EMA, eurordis and patient organisations, amongst others. Although funding is organised around three projects, the scientists involved collaborate closely to ensure that work progresses efficiently and without duplication. Some pairs of projects have members in common, as do project advisory boards, and representatives of the three projects meet regularly at conferences and scientific meetings, where current and planned research directions are presented and discussed. In some cases, this has led to closer links between projects with groups comprising scientists from more than one of the funded projects collaborating on a specific topic.

## Conclusion

The three projects address open statistical methodological design and analysis problems for clinical trials in small population groups, encompassing areas like rare diseases in paediatrics, nephrology, etc. and as well as subgroups of populations, and individualised therapy evaluation.

The description above shows the wide spread and interwoven structure of the research, which has to be addressed. Obviously, additional research will be stimulated by the investigations. The main gain will be that new methods are available with proven efficiency for small population clinical trials. The derived methods will serve as a basis for small population clinical trials in the future.

This paper aims to raise awareness of the ongoing research and stimulate other groups to work on statistical methodology on design and analysis of clinical trials in small populations. Having well informed researchers, physicians and biostatisticians will result in the use of more efficient methods to conduct a clinical trial in small population groups and thus bring approved treatments faster to our patients.

## Abbreviations

ASTERIX, Advances in Small Trials dEsign for Regulatory Innovation and eXcellence; EMA, European Medical Agency; EU, European Union; EURORDIS, European Organisation for Rare Diseases; FDA, U.S. Food and Drug Administration; IDeAl, Integrated Design and Analysi of small population group trials; InSPiRe, innovative methodology for small populations research; PD, Pharmacodynamic; PK, Pharmacokinetic; pocri, Patient-Centered Outcomes Research institute
